# Molecular Targets of Oxidative Stress: Focus on the Nrf2 Signaling Pathway in Health and Disease

**DOI:** 10.3390/antiox13030262

**Published:** 2024-02-21

**Authors:** Marcel Bonay

**Affiliations:** 1 UVSQ, INSERM END-ICAP, Université Paris-Saclay, 78000 Versailles, France; marcel.bonay@aphp.fr; Tel.: +33-170-429-415; 2 Service de Physiologie-Explorations Fonctionnelles Bi-Sites, Hôpitaux Ambroise Paré et Bicêtre, Assistance Publique-Hôpitaux de Paris, 92104 Boulogne, France

## 1. Introduction

Oxidative stress, known to increase the risk of multiple metabolic and chronic disorders or cancer development, is defined as an imbalance between the production of reactive oxygen species (ROS) and the capacity of antioxidants to counteract the deleterious effects of oxidants. To regulate the oxidation/reduction (redox) balance, numerous antioxidant enzymes and nonenzymatic antioxidants exist. Free radicals activate transcription factors to promote antioxidant production and mitochondrial biogenesis. One of these transcription factors, nuclear factor erythroid 2-related factor 2 (Nrf2), is a master regulator of antioxidant and anti-inflammatory responses. Indeed, Nrf2 contributes to redox balance by initiating the transcription of hundreds of genes involved in antioxidant and cytoprotective responses. Better understanding of the molecular targets of oxidative stress and their interaction with the Nrf2 signaling pathway would strengthen the relevance of their preventive or therapeutic use in health and diseases.

For this Special Issue, researchers were invited to submit original articles or review articles on different aspects of the modulation of oxidative stress in animal models or in humans. The topics included focused on biological and physiological effects of the Nrf2 signaling pathway in chronic diseases or their prevention.

## 2. An Overview of Published Articles

Four original articles and three reviews fell under the neuroscience theme and/or focused on neurologic disorders.

Amyotrophic lateral sclerosis is a progressive and severe disease caused by the degeneration of motor neurons; however, available treatments have only limited clinical benefits. Jiménez-Villegas et al. (contribution 1) realized targeted transcriptional profiling in leukocytes from patients with hexanucleotide expansion located in the first intron of the C9orf72 gene, a genetic alteration observed in 40% of familial amyotrophic lateral sclerosis (ALS) patients, and found an altered expression of 10 redox genes compared with healthy controls. In an in vitro model that reproduced toxic mechanisms attributed to C9orf72 pathology, Nrf2 activation by dimethyl fumarate was shown to protect motor-neuron-like hybrid cells against dipeptide repeat toxicity. This study highlights Nrf2 as a potential therapeutic target for ALS patients with C9orf72 gene expansion repeats.

In the treatment of other neurological disorders without therapeutic resources apart from mechanical ventilation, high spinal cord injuries (SCIs) induce the deafferentation of phrenic motoneurons, leading to respiratory muscle paralysis. Michel-Flutot et al. (contribution 2) assessed the antioxidant response in phrenic motoneurons involving the AMPK–Nrf2 signaling pathway following C2 spinal cord lateral hemi-section in rats, and found a reduced expression of phosphorylated AMPK and Nrf2 at one hour post injury followed by a rebound of expression at one day post injury. In the total spinal cord around phrenic motoneurons, increases in phosphorylated AMPK and Nrf2 occurred at three days post injury, showing the differential antioxidant responses between phrenic motoneurons and other cell types. The modulation of the AMPK–Nrf2 signaling pathway could improve the antioxidant response and help in spinal rewiring to these deafferented phrenic motoneurons in patients with high SCIs.

Neuroinflammation and failing redox homeostasis might be involved in the pathophysiology of neurological sequelae in long-COVID. Affecting nearly 65 million people worldwide, long-COVID is the subject of growing research [1]. Ercegovac et al. (contribution 3) investigated whether variations in antioxidant genetic profile were associated with neurological sequelae in long-COVID. Neurological examination and antioxidant genetic profile (SOD2, GPXs, and GSTs) determination, as well as genotype analysis of the key redox-sensitive transcription factors Nrf2 and ACE2, which affects binding and internalization of the coronavirus, were conducted in 167 COVID-19 patients. They found that individuals carrying the GSTP1 Val or GSTO1 Asp allele exhibit lower odds of long-COVID myalgia development, both independently and in combination. Furthermore, the combined presence of GSTP1 Ile and GSTO1 Ala alleles exhibited cumulative risk regarding long-COVID myalgia in carriers of the combined GPX1LeuLeu/GPX3CC genotype. Moreover, individuals carrying the combined GSTM1-null/GPX1LeuLeu genotype were more prone to developing long-COVID “brain fog”, while probability was further enhanced if the Nrf2 A allele was also present. The fact that certain genetic variants of antioxidant enzymes affect the probability of long-COVID manifestations further highlights the involvement of genetic susceptibility, both when SARS-CoV-2 infection is initiated in the host cells, but also months later.

The P2X7 receptor (P2X7R) is a cation-permeable ATP ligand-gated ion channel; its activation is involved in neuronal excitability, neuroinflammation, and functions of astrocytes and microglia. Lee et al. (contribution 4) assessed responses to LPS in the mouse hippocampus in vivo and showed that P2X7R increases LPS-induced neuroinflammation by leading to Nrf2 degradation, aberrant glutamate–glutamine cycle activity, and impaired cystine/cysteine uptake, which inhibit glutathione biosynthesis. They suggest that the targeting of P2X7R, which would exert nitrosative stress with iNOS in a positive feedback manner, may be one of the most important therapeutic strategies against nitrosative stress under pathophysiological conditions.

Neurodegenerative diseases, such Alzheimer’s disease and Parkinson’s disease, represent a major health problem, with millions of people affected worldwide; these conditions are leading causes of disability [2]. In their review, Amoroso et al. (contribution 5) described the implication of oxidative stress and Nrf2 activation in neurodegenerative diseases. The potential therapeutic interests of small molecules inducing Nrf2, natural Nrf2 activators, and multitargeting Nrf2 activators were discussed.

In another review, Sani et al. (contribution 6) highlighted the role of Nrf2 in depression. Most studies were performed in murine models of depression, and indicated that the Nrf2–antioxidant pathway repaired neuroinflammation and restored behavioral test performance. Although Nrf2 might represent a potential therapeutic target, the authors emphasized that animal models of depression are difficult to translate to humans.

Genetic diversity and interindividual differences in post-translational modifications might be critical for developing oxidative-stress-related diseases, such as cancer and neurological disorders. The example of Human NAD(P)H:quinone oxidoreductase 1 (hNQO1), a multifunctional and antioxidant stress protein whose expression is controlled by the Nrf2 signaling pathway, is highlighted by Pey, A.L. (contribution 7) in an exhaustive review. 

In vitro or ex vivo models were used in five studies to assess oxidative stress and the Nrf2 pathway in human clinical disorders.

Lim et al. (contribution 8) showed that a methanol extract of Ehretia tinifolia protected mouse immortalized Kupffer cells from lipopolysaccharide (LPS)-mediated oxidative stress and excessive inflammatory responses by activating antioxidant Nrf2/HO-1 and inhibiting pro-inflammatory NF-κB and MAPKs.

Deramaudt et al. (contribution 9) highlighted the capacity of repetitive magnetic stimulation (rMS) to activate the non-canonical Nrf2 pathway, modulate macrophage function, and enhance the host’s defense against bacterial infection.

Tabolacci et al. (contribution 10) demonstrated how rutin, a bioflavonoid found in some vegetables and fruits, may play a potentially cytoprotective role against UVA-induced skin damage through a purely antiapoptotic mechanism.

Pattabiraman et al. (contribution 11) found that various microRNAs carried by exosomes of non-pigmentary ciliary epithelium subjected to acute and chronic oxidative stress can regulate Nrf2 and Keap1. They suggest that this regulation may influence trabecular meshwork health and functionality, aqueous humor drainage, intraocular pressure, and primary open-angle glaucoma pathophysiology.

Slaven et al. (contribution 12) suggest that the cellular response of human mesenchymal stem cells and human lung microvascular endothelial cells to chronic low-dose-rate gamma radiation, with a downregulation of Nrf2 and related genes, is significantly different from the cellular response to acute, high-dose-rate radiation. Instead, the primary cellular response to chronic low-dose-rate gamma radiation is hypoxia and iron deficiency, with increased hypoxia signaling and the increased activation of pathways regulated by iron deficiency.

Two original articles with in vivo models and four reviews are dedicated to the Nrf2 signaling pathway in metabolism, inflammation, and cancers.

Many studies have shown that exercise training improves skeletal muscle health via multiple adaptative pathways. Bhat et al. (contribution 13) assessed the effects of 3 weeks of treadmill exercise training in wild-type and iMS-Nrf2flox/flox inducible muscle-specific Nrf2 mice. Their results suggest a critical role of Nrf2 in the beneficial effects of skeletal muscle and adaptation to exercise training.

Inflammatory bowel diseases (IBDs), such as Crohn’s disease and ulcerative colitis, are chronic inflammatory disorders that affect the gastrointestinal tract, and their incidence and clinical severity have increased worldwide [3]. Tian et al. (contribution 14) assessed the effects of dietary glucoraphanin supplementation on mitochondrial dysfunction and oxidative stress in an acute colitis mouse model induced by dextran sulfate sodium. Glucoraphanin supplementation protected the colonic histological structure, suppressed inflammatory cytokines, reduced macrophage infiltration in colonic tissues, activated AMP-activated protein kinase (AMPK), peroxisome proliferator-activated receptor-gamma coactivator (PGC)-1α, and nuclear factor erythroid 2-related factor 2 pathways in the colonic tissues of dextran-sulfate-sodium-treated mice. They suggest that dietary glucoraphanin provides a dietary strategy to alleviate IBD symptoms.

In their review, Yan et al. (contribution 15) discussed the mechanisms of ferroptosis and focused on the regulation of ferroptosis by Nrf2. A list of some clinical applications of targeting the Nrf2 signaling pathway in the treatment of diseases, such as cancers and neurodegenerative and ischemic diseases, was presented. Ishii et al. (contribution 16) clarified how stress activated MAP kinases and cyclin-dependent kinase 5 mediated the nuclear translocation of Nrf2 via Hsp90α-Pin1-Dynein motor transport machinery. Malignant tumors often express enhanced Pin1-Hsp90α signaling pathways; therefore, the authors proposed how these systems might represent a potential therapeutic target for tumor treatments.

In their review, Xia et al. (contribution 17) explained the complex interaction between Nrf2, oxidative stress, lipid metabolism, insulin signaling, and chronic inflammation in obesity. Finally, Sabatino, L. (contribution 18) described the main aspect of thyroid hormone signaling and depicted the role of Nrf2 in oxidant–antioxidant homeostasis in the thyroid hormones system.

## 3. Conclusions

Most of the studies presented in this Special Issue were dedicated to multiple metabolic and chronic disorders or cancer development. They contribute to improving understanding of the molecular targets of oxidative stress and their interaction with the Nrf2 signaling pathway in some oxidative-stress-related diseases.
